# ﻿Integrative taxonomy and phylogenetic analyses of two *Ceratomyxa* species (Myxozoa, Ceratomyxidae) from the China Sea, including a new species description

**DOI:** 10.3897/zookeys.1250.149263

**Published:** 2025-09-02

**Authors:** Chengzhong Yang, Yuting Yang, Xue Chen, Shenghua Yang, Yang Zhou, Honggang Ma, Yang Liu, Yuanjun Zhao

**Affiliations:** 1 Key Laboratory of Evolution & Marine Biodiversity (Ministry of Education), and Institute of Evolution & Marine Biodiversity, Ocean University of China, Qingdao 266003, China Chongqing Normal University Chongqing China; 2 Animal Biology Key Laboratory of Chongqing Education Commission of China, Chongqing Key Laboratory of Conservation and Utilization of Freshwater Fishes, College of Life Sciences, Chongqing Normal University, Chongqing 401331, China Ocean University of China Qingdao China; 3 School of Marine Science and Engineering, Qingdao Agricultural University, Qingdao, Shandong 266237, China Qingdao Agricultural University Qingdao China

**Keywords:** *Ceratomyxa dactyloptena* sp. nov., intraspecific variation, morphology, phylogeny, rDNA

## Abstract

Two species of *Ceratomyxa* (Myxozoa: Ceratomyxidae) from teleosts in the China Sea are investigated using an integrative approach which combines morphological characteristcs, parasitic traits, and molecular data including small subunit ribosomal DNA (SSU rDNA) and internal transcribed spacer (ITS) regions. A novel species, *Ceratomyxa dactyloptena***sp. nov.**, was identified from the gallbladder of Oriental flying gurnard *Dactyloptena orientalis* Cuvier, 1829, while *Ceratomyxa siganicola* Zhang, Zhao, Yang & Yang, 2019 was found in the gallbladder of Mottled spinefoot *Siganus fuscescens* Houttuyn, 1782 and Mozambique tilapia *Oreochromis mossambicus* Peters, 1852. Mature myxospores of *C. dactyloptena***sp. nov.** exhibit a crescent shape in sutural view, with dimensions of 30.0 ± 1.3 (27.2–32.8) µm in thickness, 6.4 ± 0.4 (5.7–7.0) µm in length, and a posterior angle of 152.9 ± 3.7° (146.2–158.9°). Pyriform polar capsules measured 2.9 ± 0.3 (2.3–3.3) µm long and 2.4 ± 0.3 (1.7–2.8) µm wide, with polar filaments coiled in 3–4 turns. The SSU rDNA sequence of *C. dactyloptena***sp. nov.** was distinct from all known myxosporeans, showing the highest similarity (87.9%) and the shortest genetic distance (0.1209) with *C. filamentosi* Kalatzis, Kokkari & Katharios, 2013. Molecular analyses revealed intraspecific variation among *C. siganicola* strains, with ITS rDNA being more sensitive than SSU rDNA in detecting these differences. Phylogenetic analysis placed *C. dactyloptena***sp. nov.** in a clade with *C. oxycheilinae* Heiniger, Gunter & Adlard, 2008, while all *C. siganicola* strains clustered together, forming a sister clade to *C. barnesi* Gunter, Whipps & Adlard, 2009. Furthermore, the phylogenetic results indicated that ceratomyxid diversification is influenced not only by host specificity but also by geographic distribution.

## ﻿Introduction

Myxozoans represent a highly diverse and globally distributed group of microscopic, spore-forming endoparasites, with over 2,600 described species, predominantly infecting fish hosts (Okamura et al. 2018; Zatti et al. 2022). Among these, the genus *Ceratomyxa* Thélohan, 1892, is notably prolific, encompassing over 300 species—accounting for approximately 12% of all known myxosporeans (~300 out of ~2600 species) (Eiras 2006; Eiras et al. 2018; Rocha et al. 2023). *Ceratomyxa* species are characterized by their elongate, crescent-shaped or arcuate myxospores, with shell valves that often exhibit conical extensions beyond the myxospore’s axial diameter. The myxospores contain subspherical polar capsules with capsular foramina located near the suture line at the anterior pole, although in some rare cases, the polar capsules open on opposite sides of the suture (Lom and Dyková 1992). Most *Ceratomyxa* species are coelozoic parasites that inhabit the gallbladders of marine teleosts. A smaller subset has been reported from freshwater fishes, and, more rarely, from of elasmobranchs. Among the latter, notable examples include *C. carcharhini* Gleeson & Adlard, 2011 and *C. melanopteri* Gleeson & Adlard, 2011, both described from *Carcharhinus melanopterus* Quoy & Gaimard, 1824, as well as *C. negaprioni* Gleeson & Adlard, 2011 from *Negaprion acutidens* Rüppell, 1837 (Eiras 2006; Gleeson and Adlard 2011; Eiras et al. 2018; Surendran et al. 2024). To enhance taxonomic precision and reduce potential misidentifications associated with morphology-based identification alone, an integrative approach incorporating both morphological and molecular data is recommended for the identification of ceratomyxids (Atkinson et al. 2015; Zhang et al. 2019; Huang et al. 2020). Nevertheless, most ceratomyxid classifications remain morphology-based, and only around 90 *Ceratomyxa* species have been characterized to date using SSU rRNA gene data, with fewer studies utilizing additional molecular markers (Eiras 2006; Gunter and Adlard 2010; Yang et al. 2023).

The marine ecosystems of the China Sea support a rich diversity of fish species, yet studies on their myxozoan parasites remain scarce (Yang et al. 2023). In a recent survey of myxozoan diversity in this region, we identified two *Ceratomyxa* species through an integrative approach that combining morphological characteristics, parasitic traits, and molecular data.

## ﻿Materials and methods

### ﻿Sample collection and morphological analysis

**Figure 1.**
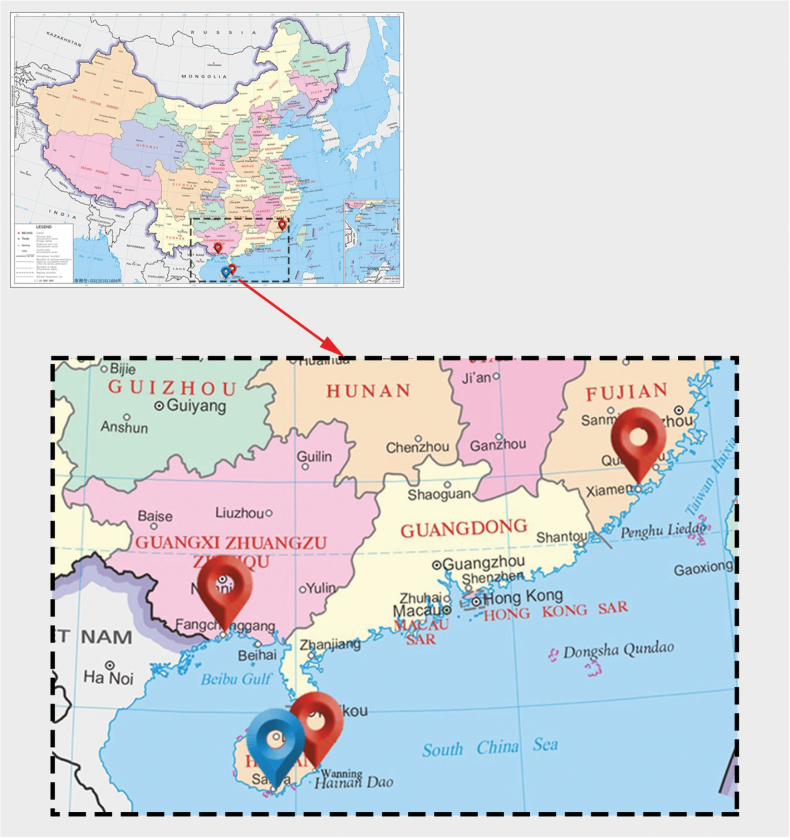
Sampling localities for hosts of *Ceratomyxa dactyloptena* sp. nov. (blue map pin) and *Ceratomyxa siganicola* (red map pins).

Sixty specimens of Mottled spinefoot *Siganus fuscescens* Houttuyn, 1782, 18 specimens of Mozambique tilapia *Oreochromis mossambicus* Peters, 1852, and 14 specimens of Oriental flying gurnard *Dactyloptena orientalis* Cuvier, 1829 were collected from the China Sea (sampling sites shown in Fig. 1) and subsequently transported to the laboratory for parasitological examination. Comprehensive examinations were conducted on various organs—including the skin, fins, gills, muscles, hepatopancreas, intestines, spleen, heart, gallbladder, and urinary bladder—as well as body fluids such as bile, blood, and urine, for detecting myxozoan parasites. Myxospores identified were subjected to both morphological and molecular analysis. Species identification and specimen processing followed established protocols (Zhao et al. 2001). Fresh myxospores were observed under a Leica DM6000B microscope at 1000× magnification, with morphometric data derived from over 25 mature myxospores per *Ceratomyxa* species. Spore dimensions were measured using Photoshop based on light microscope images, with calibration performed using the scale bar present in each image. Measurements are presented in micrometers (μm), expressed as the arithmetic mean ± standard deviation with ranges indicated in parentheses. Line drawings were generated using CorelDRAW v. 11.0 to illustrate morphological characteristics.

The morphological comparison between the new species and other ceratomyxids was conducted based on spore morphology, with reference to all valid species of the genus *Ceratomyxa*. Particular attention was given to species infecting hosts of identical or related taxonomy and occurring in the same or geographically similar regions.

Morphological variations between *C. siganicola* Zhang, Zhao, Yang & Yang, 2019 strains and the newly identified *C. dactyloptena* sp. nov. were statistically evaluated using a non-parametric Mann–Whitney *U* test. To further explore interstrain relationships and morphological variations, a principal component analysis (PCA) was conducted on all new morphological data using PAST3 v. 2.10 (Hammer et al. 2001).

### ﻿DNA extraction, amplification, and sequencing

Myxospores isolated from host bile were preserved in 95% ethanol. Genomic DNA was extracted using the DNeasy Blood and Tissue Kit (QIAGEN GmbH, Hilden, Germany), following the manufacturer’s protocol. Amplification of the small subunit ribosomal DNA (SSU rDNA) was performed in a 25-μL PCR reaction containing 0.5 μM of each primer—ERIB1 (5′-ACCTGGTTGATCCTGCCAG-3′) and ERIB10 (5′-CTTCCGCAGGTTCACCTACGG-3′) (Barta et al. 1997), as well as CERAss1-F (5′-CGCTCCAAGTGAGTGCCATC-3′) / CERAss1-R (5′-ACCTGTTATTGCCACGCTTCC-3′) and CERAss2-F (5′-GAAGCGTGGCAATAACAGGTC-3′) / CERAss2-R (5′-AGAGGCAGAGACGTATTCAACA-3′), which were newly designed using Primer6, were used in combination with 2.5 mM MgCl_2_, 2.5 mM of each dNTP, 20 ng of genomic DNA, and 1.5 U of Taq Ex DNA polymerase (TaKaRa, Otsu, Japan). The PCR protocol included an initial denaturation at 94 °C for 5 min, followed by 35 cycles of 94 °C for 20 s (denaturation), 55 °C for 20 s (annealing), and 72 °C for 2 min (extension), with a final extension at 72 °C for 10 min. To amplify the Internal Transcribed Spacer (ITS) region, the primers CERA-F (5′-TCGACTCTGCCATACTTGCA-3′) and CERA-R (5′-GTGTATGCAAGCACAACTTTGACAA-3′) were specifically designed using Primer6 and employed under identical PCR conditions. The PCR products were separated by agarose gel electrophoresis at 140 V for 20 min, and fragments were purified using the DNA Agarose Gel Extraction Kit (Omega Bio-Tek, Norcross, GA, USA). The purified amplicons were ligated into a pMD19-T vector (TaKaRa, Otsu, Japan), and *Escherichia coli* strain DH5a was transformed with this construct. Eight transformants were grown in culture, plasmids purified, and four clones were sequenced using an ABI Prism 377 DNA Sequencer. The SSU rDNA and ITS rDNA sequences of myxospores were assembled using ContigExpress software and were submitted to the National Center for Biotechnology Information (NCBI) nucleotide database for public access.

### ﻿Molecular and phylogenetic analyses

To evaluate the genetic similarities and genetic distances between the SSU rDNA sequences obtained in this study and other *Ceratomyxa* taxa, we retrieved all available sequences of ceratomyxids from the NCBI database. To avoid redundancy, only one representative sequence per species was included in the analyses. Pairwise sequence similarity was performed using WATER program of the EMOSS suit (Madeira et al. 2024) and genetic distances (*p*-distance) were calculated in MEGA11 (Tamura et al. 2021).

A phylogenetic tree was constructed from a total of 98 SSU rDNA sequences, including three newly obtained sequences representing two genotypes of *Ceratomyxa siganicola* and one of *C. dactyloptena* sp. nov., along with closely related sequences retrieved from GenBank. *Enteromyxum scophthalmi* Palenzuela, Redondo & Alvarez-Pellitero, 2002 (AF411335) and *Enteromyxum fugu* (Tin Tun, Yokoyama, Ogawa & Wakabayashi, 2000) comb. nov. by Yanagida, Nomura, Kimura, Fukuda, Yokoyama & Ogawa, 2004 (AY520573) were included as outgroups due to their status as early-branching relatives of *Ceratomyxa* (Gunter et al. 2009). Multiple sequence alignments were performed using Clustal X with default parameters (Thompson 1997). Highly variable, misaligned, or rapidly evolving positions were removed using Gblocks (Castresana 2000). The final alignment length is 1708 bp. Phylogenetic trees were constructed via both Bayesian Inference (BI) and Maximum Likelihood (ML) methods. BI analysis was conducted with MrBayes v. 3.1.2 (Ronquist and Huelsenbeck 2003), applying the optimal evolutionary model GTR+I+G as determined by Modeltest v. 3.7 (Posada and Crandall 1998) based on the Akaike information criterion. Four independent Markov Chain Monte Carlo (MCMC) runs were performed for 3 million generations, with tree sampling every 200 generations and the initial 25% discarded as burn-in. ML analysis was conducted on the CIPRES portal (https://www.phylo.org/) using RA×ML (Stamatakis 2014) with the GTR+gamma model, and bootstrap support values were calculated from 1,000 replicates. Additionally, an unrooted neighbor-joining (NJ) tree based on the ITS rDNA region of five *C. siganicola* strains was constructed using MEGA 11 (Tamura et al. 2021). The phylogenetic trees were visualized and refined with FigTree v. 1.4.2 and Photoshop CS6.

## ﻿Results

### ﻿Taxonomic summary

**Phylum Cnidaria Hatschek, 1888**

**Class Myxozoa Grassé, 1970**

**Subclass Myxosporea Bütschli, 1881**

**Order Bivalvulida Shulman, 1959**

**Family Ceratomyxidae Doflein, 1899**

**Genus *Ceratomyxa* Thélohan, 1892**

#### ﻿ Ceratomyxa dactyloptena sp. nov.

Taxon classificationAnimaliaBivalvulidaCeratomyxidae

5831EFC0-FFEB-5700-B1CB-3C64023D7CEF

https://zoobank.org/648669E0-FADE-4348-BA2C-06DD9AA2A508

##### Type host.

Oriental flying gurnard *Dactyloptena orientalis* Cuvier, 1829.

##### Type locality.

Coastal waters near Sanya, South China Sea (18°14'32"N, 109°30'31"E).

##### Infection site.

Gallbladder.

##### Prevalence.

42.9% (6/14). The infected hosts included 5 adults and 1 subadult.

##### Date of sampling.

15 August 2016.

##### Type material.

Syntypes (mounted in glycerin-alcohol-formalin; accession numbers SY2016081501) and DNA samples (accession numbers SY2016081501dna) were deposited in collection center of Animal Biology Key Laboratory of Chongqing Education Commission of China, Chongqing, PR China.

##### Pathology.

The parasite was found in the gallbladder of the host with no obvious pathological sign being observed. The damage to the host is unknown.

##### Etymology.

The species epithet *dactyloptena* refers to the genus of the type host, *Dactyloptena*.

##### Description.

**Figure 2.**
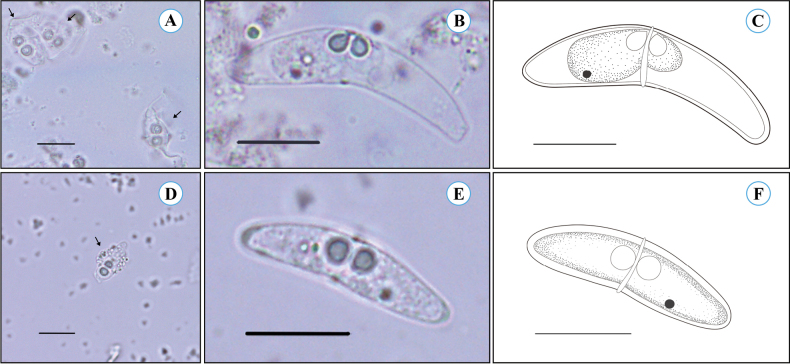
Light microscope images and line drawings of ceratomyxids from this study. **A–C.** Immature myxospores (black arrows), mature myxospore, and line drawing of *Ceratomyxa dactyloptena* sp. nov., respectively **D–F.** Immature myxospore (black arrow), mature myxospore, and line drawing of *Ceratomyxa siganicola*, respectively.

Immature myxospores at the vegetative stage were observed, displaying irregular shapes with two faintly discernible shell valves, and two polar capsules were distinctly visible (Fig. 2A). Mature myxospores were crescentic in sutural view, characterized by a slightly concave posterior margin and arched anterior, typical of the genus *Ceratomyxa*. The two shell valves were smooth, with one valve slightly longer than the other, joined by a thin, straight sutural line passing between the two polar capsules. Polar capsules were pyriform, equal in size, with their tapered ends directed toward the anterior top of the myxospore (Fig. 2B, C). Mature myxospores (*N* = 30) measured 30.0 ± 1.3 (27.2–32.8) µm in thickness and 6.4 ± 0.4 (5.7–7.0) µm in length, with a posterior angle of 152.9 ± 3.7° (146.2–158.9°). Two polar capsules (*N* = 60) were 2.9 ± 0.3 (2.3–3.3) µm in length and 2.4 ± 0.3 (1.7–2.8) µm in width, with polar filaments coiled in 3–4 turns.

##### Remarks.

*Ceratomyxa dactyloptena* sp. nov. is the only ceratomyxid identified from *D. orientalis* and morphologically resembling *C. drepanopsettae* Awerinzew, 1908, *C. macapaensis* Bittencourt, 2022, and *C. orientalis* Dogiel, 1948. *Ceratomyxa drepanopsettae* can be readily distinguished from *C. dactyloptena* sp. nov. by its substantially longer and thicker myxospores (Table 1). Its polar capsules are also larger, and notably spherical, contrasting with the pyriform polar capsules observed in *C. dactyloptena* sp. nov. (Table 1). Additionally, *C. drepanopsettae* was described from *Pleuronichthys cornutus* Temminck & Schlegel, 1846 collected in the Yellow Sea, off Qingdao, China, whereas *C. dactyloptena* sp. nov. infects *D. orientalis* and was identified from the South China Sea, off Sanya, China. *Ceratomyxa macapaensis*, a freshwater species and differs significantly from the new species by its smaller myxospores and smaller polar capsules (Table 1). Although both species possess pyriform polar capsules, their environmental origins clearly separate them: *C. macapaensis* was discovered in the Piririm River (Brazil), parasitizing *Mesonauta festivus* Heckel, 1840, while *C. dactyloptena* sp. nov. is marine and occurs in Chinese coastal waters. *Ceratomyxa orientalis* possesses considerably longer and thicker myxospores compared to the new species (Table 1). Its polar capsules are also slightly larger (Table 1). Furthermore, *C. orientalis* was described from *Sardinops sagax* Jenyns, 1842 in the Sea of Japan (Russia) and clearly differs in both host and locality from *C. dactyloptena* sp. nov.

**Table 1.** Comparative morphometrics of *Ceratomyxa dactyloptena* sp. nov. with similar species. ND, data not available.

Species	Myxospore size (length × thickness; µm)	Polar capsule size (length × width; µm)	Posterior angle (°)	Polar capsule shape	Host	Locality	Reference
*C. dactyloptena* n. sp.	6.4 ± 0.4(5.7–7.0) × 30.0 ± 1.3(27.2–32.8)	2.9 ± 0.3 (2.3–3.3) × 2.4 ± 0.3 (1.7–2.8)	152.9 ± 3.7 (146.2–158.9)	Pyriform	*Dactyloptena orientalis* Cuvier, 1829	South China Sea, off Sanya, China	**Present study**
*C. drepanopsettae* Awerinzew, 1908	11.3(11–12) × 56.8 (52–62)	4.3(4.0–4.5)	ND	Spherical	*Pleuronichthys cornutus* Temminck & Schlegel, 1846	Yellow Sea, off qingdao, China	Zhao and Song 2003
*C. macapaensis* Bittencourt da Silva, Hamoy, de Carvalho, da Silva, Videira, Carvalho & Matos, 2022	4.2 ± 0.5 × 22.75 ± 0.3	1.86 ± 0.3 × 1.63 ± 0.1	ND	Pyriform	*Mesonauta festivus* Heckel, 1840	Piririm River, Amapá, Brazil	Bittencourt et al. 2022
*C. orientalis* Dogiel, 1948	7–11 × 33–72	3–3.3	ND	ND	*Sardinops sagax* Jenyns, 1842	Sea of Japan, Russia	Eiras 2006;
*C. qingdaoensis* Zhao, Al-Farraj, Al-Rasheid & Song, 2015	6.5 ± 0.5 (6.0–7.0) × 46.0 ± 2.8 (42.0–48.0)	2.8 ± 0.2 (2.5–3.0)	162–175	Spherical	*Argyrosomus argentatus* Houttuyn, 1872	Yellow Sea, off Qingdao, China	Zhao et al. 2015
*C. saurida* Zhao, Al-Farraj, Al-Rasheid & Song, 2015	9.7 ± 0.5 (9.0–10.5) × 42.7 ± 2.5 (39.5–47)	3.6 ± 0.4 (3.0–4.0)	130–165	Spherical	*Saurida elongata* Temminck & Schlege, 1846	Yellow Sea, off Qingdao, China	Zhao et al. 2015
*C. hemitriptera* Zhao, Al-Farraj, Al-Rasheid & Song, 2015	9.2 ± 0.3 (8.8–9.5) × 83.0 ± 3.3 (80.0–88.9)	4.8 ± 0.2 (4.0–5.0)	162–170	Spherical	*Hemitripterus villosus* Pallas, 1814	Yellow Sea, off Qingdao, China	Zhao et al. 2015

Among all valid *Ceratomyxa* species recorded in China, *C. qingdaoensis* Zhao, Al-Farraj, Al-Rasheid & Song, 2015, *C. saurida* Zhao, Al-Farraj, Al-Rasheid & Song, 2015, and *C. hemitriptera* Zhao, Al-Farraj, Al-Rasheid & Song, 2015 share some morphological resemblance to *C. dactyloptena* sp. nov. However, the new species can be reliably distinguished from these species based on a combination of spore dimensions, polar capsule characteristics, host, and locality data. The myxospore length of *C. dactyloptena* sp. nov. is nearly identical to that of *C. qingdaoensis*, yet the latter possesses markedly greater spore thickness (Table 1). The polar capsule length of *C. qingdaoensis* is comparable to that of the new species, but its polar capsules are spherical, whereas those of the new species are pyriform (Table 1). Additionally, *C. qingdaoensis* exhibits a larger posterior angle than *C. dactyloptena* sp. nov. and occurs in a different host (*Argyrosomus argentatus* Houttuyn, 1872) and locality (Yellow Sea, off Qingdao, China). *Ceratomyxa saurida* can be distinguished from *C. dactyloptena* sp. nov. by its larger myxospores and longer polar capsules (Table 1). Similar to *C. qingdaoensis*, the polar capsules of *C. saurida* are spherical, contrasting with the pyriform capsules of the new species. The posterior angle of *C. saurida* is slightly more variable and overlaps partially with that of the new species, but its host (*Saurida elongata* Temminck & Schlege, 1846) and distribution (Yellow Sea, off Qingdao, China) are distinct (Table 1). *Ceratomyxa hemitriptera* is readily separated from *C. dactyloptena* sp. nov. by having considerably thicker myxospores and larger polar capsules (Table 1). The myxospore length is also greater (Table 1). As with the other two species, *C. hemitriptera* has spherical polar capsules, while those of *C. dactyloptena* sp. nov. are pyriform. Moreover, *C. hemitriptera* infects a different host (*Hemitripterus villosus* Pallas, 1814) and is found in a different geographic location (Yellow Sea, off Qingdao, China).

#### ﻿ Ceratomyxa siganicola

Taxon classificationAnimaliaBivalvulidaCeratomyxidae

Zhang, Zhao, Yang & Yang, 2019

4EA8B1AB-1592-52A7-8494-8A5FA49F1979

##### Type host.

Mottled spinefoot *Siganus fuscescens* Houttuyn, 1782 (Perciformes, Siganidae) for strain 1 (thereafter called S1 for short), strain 2 (thereafter called S2 for short), strain 3 (thereafter called S3 for short) and strain 5 (thereafter called S5 for short).

##### Other host.

Mozambique tilapia *Oreochromis mossambicus* Peters, 1852 (Perciformes: Cichlidae) for strain 4 (thereafter called S4 for short).

##### Infection site.

Gallbladder.

##### Date and localities of sampling.

April 24, 2021 in coastal waters of Xiamen, East China Sea, China (24°28'2"N, 118°4'41"E) for S1; May 1, 2021 in coastal waters of Xiamen, East China Sea, China (24°27'53"N, 118°4'47"E) for S2; November 9, 2020 in coastal waters of Fangchenggang, South China Sea, China (21°41'8"N, 108°21'25"E) for S3; November 9, 2020 in coastal waters of Fangchenggang, South China Sea, China (21°41'27"N, 108°21'3"E) for S4; August 5, 2021 in coastal waters of Wanning, South China Sea, China (18°45'3"N, 110°27'49"E) for S5.

##### Prevalence.

66.7% (8/12) for S1; 33.3% (5/15) for S2; 25.0% (2/8) for S3; 33.3% (6/18) for S4; 40.0% (10/25) for S5.

##### Pathology.

The parasite is found in the gallbladder of the host. No obvious pathological sign was observed, so the extent of damage to the host remains unknown.

##### Deposition of materials.

Specimens of *C. siganicola* (mounted in glycerin-alcohol-formalin; accession number XM2021042401 for S1, XM2021050101 for S2, GX2020110901 for S3, GX2020110902 for S4, HN2021080501 for S5) and DNA (accession number XM-2021042401dna for S1, XM-2021050101dna for S2, GX-2020110901dna for S3, GX-2020110902dna for S4, HN-2021080501dna for S5) were deposited in the collection center of Animal Biology Key Laboratory of Chongqing Education Commission of China, Chongqing, PR China.

##### Description.

Immature myxospores in the vegetative stage displayed irregular shapes with two faintly discernible shell valves. Despite the irregular morphology, two polar capsules were clearly visible (Fig. 2D). Mature myxospores exhibited morphological traits consistent with the genus *Ceratomyxa*. In side sutural view, myxospores were boat-shaped, with two smooth, symmetrical shell valves terminating in straight, blunt ends. The sutural line was straight and distinct, dividing the myxospore into two equal valves (Fig. 2E, F). Measurements of mature myxospores (*N* = 30) revealed a thickness of 19.2 ± 5 (17.7–21.5) µm and a length of 4.8 ± 0.6 (3.8–6.0) µm. The posterior angle was slightly concave, measuring 171.8 ± 3.3° (162.7–179.4°). Each myxospore contained two spherical, equal-sized polar capsules with a diameter of 2.5 ± 0.3 (2.0–3.2) µm and polar filaments with 3–4 coils. The morphological features observed in this sample align with previous descriptions of *C. siganicola*, particularly with the original description by Zhang et al. (2019).

### ﻿Morphometric analyses

**Figure 3.**
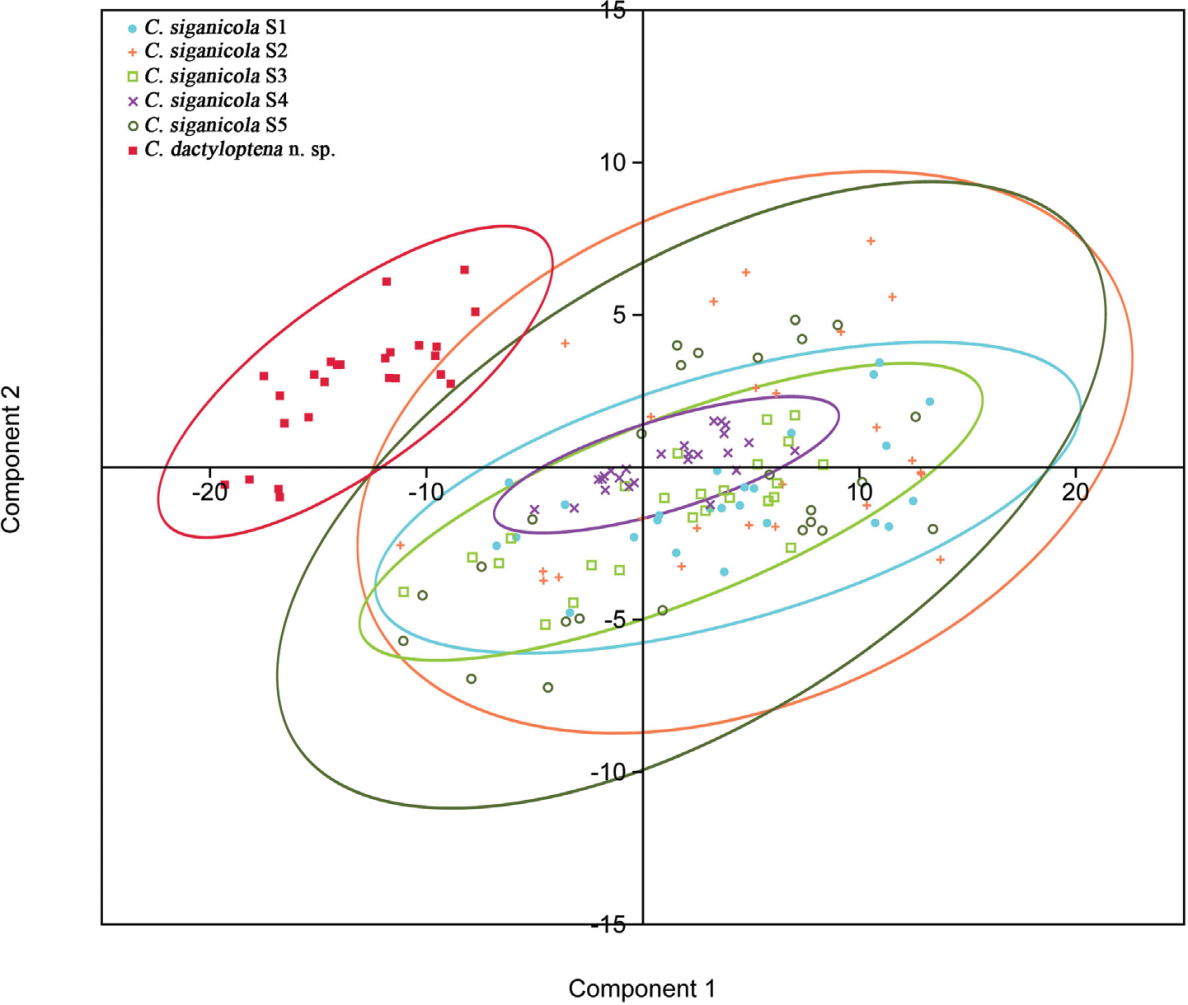
Principal component analysis (PCA) of ceratomyxids, including *Ceratomyxa dactyloptena* sp. nov. and five strains (S1–S5) of *Ceratomyxa siganicola*.

The PCA indicated minimal morphological variation among the five strains of *C. siganicola*, while clearly differentiating *C. siganicola* from *C. dactyloptena* sp. nov. (Fig. 3). The results of the Mann–Whitney *U* test further confirmed no statistically significant differences among the five strains of *C. siganicola* (*P* > 0.05), while revealing significant differences between *C. siganicola* and *C. dactyloptena* sp. nov. (*P* < 0.05).

### ﻿Molecular analyses

A partial SSU rDNA sequence of *C. dactyloptena* sp. nov., 1719 bp in length, was successfully amplified and sequenced from the type host and has been deposited in GenBank under accession number PP535027. Molecular analyses revealed that this sequence exhibited the highest similarity (87.9%) and the shortest genetic distance (*p* = 0.1209) to *C. filamentosi* Kalatzis, Kokkari & Katharios, 2013 (JX869944), followed by *C. cretensis* Kalatzis, Kokkari & Katharios, 2013 (JX869942) with 86.4% similarity and a genetic distance of 0.1302. Among species that share morphological resemblance with *C. dactyloptena* sp. nov., only *C. saurida* and *C. macapaensis* possess a publicly available SSU rDNA sequence. The sequence similarity and genetic distance between the new species and *C. saurida* (MT808644) were 85.7% and 0.1330, respectively, while those between the new species and *C. macapaensis* (MT939250) were 74.2% and 0.2382, respectively.

For five strains (S1–S5) of *C. siganicola*, SSU rDNA sequences were obtained with lengths of 1723, 1736, 1779, 1782, and 1785 bp, respectively. Sequence similarity among these strains ranged from 98.9% to 100.0%, with pairwise genetic distances between 0.0006 and 0.0060. These sequences have been deposited in GenBank under accession numbers PP535021 to PP535025. Comparisons with existing *C. siganicola* sequences in GenBank revealed high similarity values (98.9–100.0%) and genetic distances ranging from 0.0000 to 0.0094. Additionally, ITS rDNA sequences were obtained from the five strains, with lengths of 1479, 1485, 1467, 1492, and 1467 bp, respectively. Sequence similarities ranged from 92.7% to 100.0%, and genetic distances from 0.0000 to 0.0840. These ITS rDNA sequences have been deposited in GenBank under accession numbers PP777511 to PP777515.

**Figure 4.**
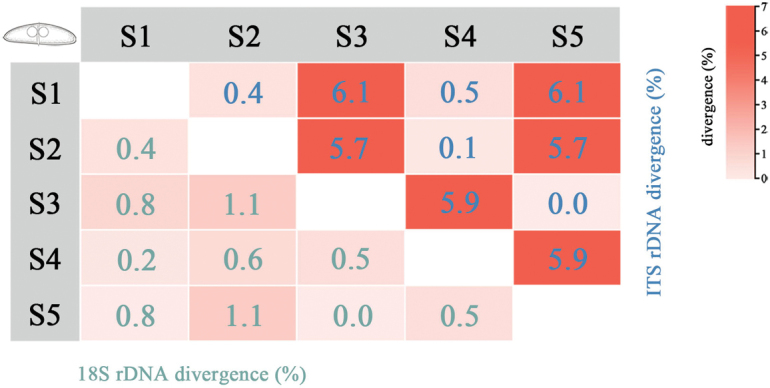
Genetic divergence among five strains of *Ceratomyxa siganicola* based SSU rDNA and ITS rDNA sequences.

Divergence analyses among strains indicated that the degree of genetic variation was dependent on the molecular marker used (Fig. 4). Among strains derived from the same host species, the average genetic divergence based on SSU rDNA was 0.7% (0.0–1.1%), whereas divergence based on ITS rDNA reached 4.0% (0.0–6.1%). Among strains from different host species, average genetic divergence was 0.45% (0.2%–0.6%) for SSU rDNA and 3.1% (0.1%–5.9%) for ITS rDNA.

### ﻿Phylogenetic analyses

**Figure 5.**
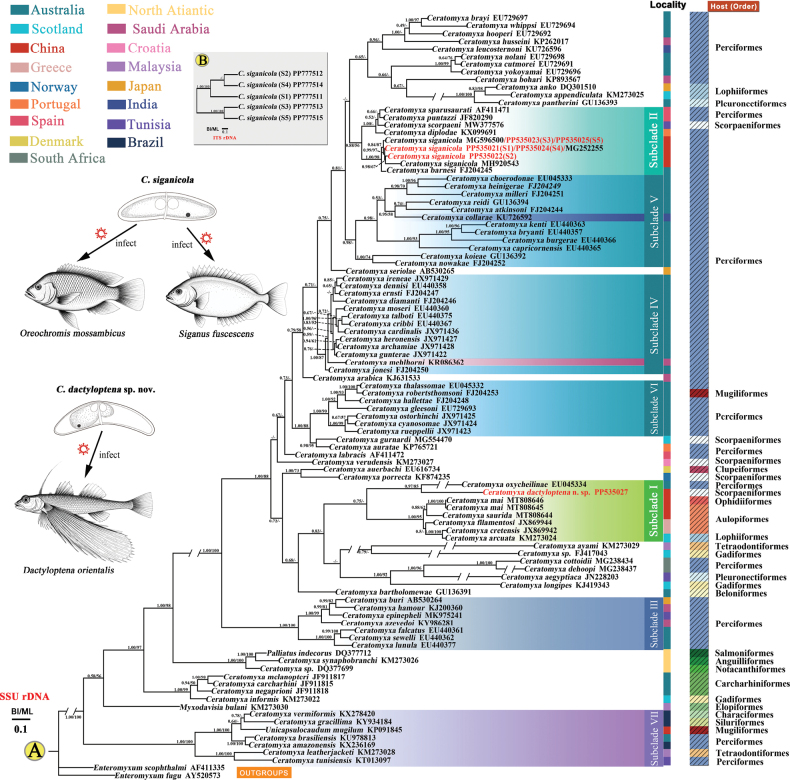
Phylogenetic relationships of *Ceratomyxa dactyloptena* n. sp. and *Ceratomyxa siganicola* based on molecular data. A. BI and ML phylogenetic tree based on SSU rRNA gene sequences of *C. dactyloptena* sp. nov., *C. siganicola*, and closely related species. Numbers given at nodes are Bayesian posterior probabilities and bootstrap support, with “-” indicating values less than 50%. “//” indicates that the branch is drawn 1/3 of the original length; B. BI and ML phylogenetic tree based on ITS rRNA gene sequences of the five strains of *C. siganicola*.

The phylogenetic trees constructed based on SSU rRNA gene sequences using Maximum-likelihood (ML) and Bayesian-inference (BI) methods exhibited consistent topologies (Fig. 5A). The phylogeny revealed that the genus *Ceratomyxa* is not monophyletic, as three non-*Ceratomyxa* species (*Palliatus indecorus* Shulman, Kovaljova & Dubina, 1979, *Myxodavisia bulani* Fiala, Hlavničková, Kodádková, Freeman, Bartošová-Sojková & Atkinson, 2015, and *Unicapsulocaudum mugilum* Yang, Zhou, Zhao, Huang & Huang, 2017) were nested within early-diverging lineages of the *Ceratomyxa* clade (Fig. 5A). Their positions disrupt the monophyly of *Ceratomyxa*, suggesting that these taxa may represent separate evolutionary lineages within a broader ceratomyxid radiation. The overall phylogeny shows a deep split within the *Ceratomyxa* clade, forming two major evolutionary lineages: (1) an early-diverging lineage (subclade VII) comprising freshwater, brackish, and marine species, and (2) a more derived and species-rich lineage made up of almost exclusively marine species. This latter group includes both the defined subclades (I–VI) and several additional *Ceratomyxa* lineages that remain unnumbered but form distinct independent branches in the tree (Fig. 5A). Within subclade VII, two internal lineages are resolved: one comprises the two marine species (*C. leatherjacketi* Fiala, Hlavničková, Kodádková, Freeman, Bartošová-Sojková & Atkinson, 2015 and *C. tunisiensis* Thabet, Mansour, Omar & Tlig-Zouari, 2016), while the other includes four freshwater (*C. vermiformis* Adriano & Okamura, 2017; *C. gracillima* Zatti, Atkinson, Maia, Bartholomew & Adriano, 2018; *C. brasiliensis* Zatti, Atkinson, Bartholomew, Maia & Adriano, 2017; *C. amazonensis* Mathews, Naldoni, Maia & Adriano, 2016) and a brackish-water species (*Unicapsulocaudum mugilum*) (Fig. 5A).

Differences in host spectrum are observed across the phylogeny: early-diverging subclades such as subclade VII, as well as a relatively basal lineage (subclade I), include species infecting hosts from diverse taxonomic groups (Characiformes, Siluriformes, Mugiliformes, Perciformes, and Tetraodontiformes for subclade VII; Perciformes, Scorpaeniformes, Ophidiiformes, Aulopiformes and Lophiiformes for subclade I), whereas species in more derived clades (e.g. subclades IV, V and VI) tend to have narrower host ranges (Perciformes for subclades IV; Perciformes for subclades V; Perciformes and Mugiliformes for subclade VI).

Phylogenetic analysis also indicated that *C. dactyloptena* sp. nov. shares the closest evolutionary relationship with *C. oxycheilinae* Heiniger, Gunter & Adlard, 2008, with both species forming a distinct subclade within an intermediate divergent lineage of the genus *Ceratomyxa* (Fig. 5A: subclade I). All strains of *C. siganicola* clustered together and were sisters to *C. barnesi* Gunter, Whipps & Adlard, 2009 within a more recently divergent lineage of *Ceratomyxa* (Fig. 5A: subclade II).

Similarly, phylogenetic trees derived from ITS rRNA gene sequences of five *C. siganicola* strains, inferred using both ML and BI methods, also displayed identical topologies (Fig. 5B). In the resulting consensus tree, the five *C. siganicola* strains were resolved into two clades: strains S2 and S4 clustered together as a sister group to S1, with these three strains forming a clade that is sister to a separate clade containing strains S3 and S5 (Fig. 5B).

## ﻿Discussion

*Ceratomyxa dactyloptena* sp. nov., the first ceratomyxid species reported to infect *Dactyloptena orientalis*, displays genus-typical morphological traits, notably a unique crescent shape and targeted parasitism of the gall bladder. Morphological characteristics distinguish it from all previously described species of the genus. Genetically, *C. dactyloptena* sp. nov. exhibits significant divergence beyond intraspecific levels when compared to all currently available SSU rDNA sequences of other myxosporeans. Although no specific benchmark exists for interspecific SSU rRNA gene variation, values ranging from 1.0% to 1.3% suggest the presence of distinct taxa, corroborated by other characteristics such as host, tissue, or morphometry (Gunter and Adlard 2009; Gunter et al. 2009; Heiniger and Adlard 2013; Yang et al. 2023).

Previous studies suggested a qualitative association between myxosporean phylogeny and the taxonomy of their hosts, with closely related myxosporeans often infecting hosts within the same taxonomic group (Gao et al. 2020; Zhang et al. 2023; Samshuri and Borkhanuddin 2024). In line with this pattern, the hosts for parasites in subclades III, IV, and V in our study are all classified within the order Perciformes (Fig. 5A). These derived lineages exhibit relatively narrow host ranges and a tendency toward host fidelity. However, not all clades follow this trend, for instance, parasites within subclade I exhibit close phylogenetic relationships, yet their hosts are assigned to distinct higher taxonomic classifications. Similarly, subclade VII contains species parasitizing both marine and freshwater fishes across a broad taxonomic spectrum (Fig. 5A). This pattern suggests a potential link between phylogenetic position and host range, where early-diverging lineages (e.g. subclades I and VII, near the root of the tree) display broader host spectra (host generalists), while later-diverging lineages (e.g. subclades IV, V, VI) tend to be host-specific. This inference raises the question of whether host specialization has evolved progressively over time within the genus, with early lineages maintaining generalist strategies and later ones exhibiting increasing specificity. Early ceratomyxids might have been generalists exploiting multiple hosts, perhaps owing to broad physiological compatibility or overlapping habitats of their hosts. Over time, lineage-specific adaptations, such as tolerance to particular bile chemistry or evasion of specific immune defenses, could have driven a shift toward narrower host use. Specializing on one host lineage can enhance parasite fitness in that niche (Kawecki 1998; Poulin 2011) but often at the cost of reduced flexibility. This evolutionary transition from generalism to specialism would explain why basal clades like I and VII infect diverse host groups, whereas derived clades like subclades IV, V, VI are restricted to closely related hosts (Fig. 5A).

In addition to these observations, we also observed that several species with close phylogenetic relationships are consistently found in the same localities. Notably, many of the ceratomyxids from Australia exhibit close phylogenetic ties, such as those within subclade VI, all of which are from Australia (Fig. 5A). Additionally, the majority of ceratomyxids in subclade IV and V are also from Australia (Fig. 5A). However, not all ceratomyxids with close phylogenetic relationships, such as those in subclade I, II, III and VII, originate from the same locality (Fig. 5A). Therefore, the phylogenetic relationships of ceratomyxids are influenced not only by host groups but also by their geographical distribution, likely resulting from the interplay of multiple evolutionary factors.

Host species, geographic isolation, and the site of parasitism are generally considered critical factors influencing population divergence (Criscione et al. 2005; Dietrich et al. 2011; Wang et al. 2019; Xiang et al. 2024). In the present study, genetic divergence analyses based on SSU rDNA and ITS rDNA revealed that *C. siganicola* has undergone a certain degree of strain differentiation. Notably, the genetic divergence among strains of *C. siganicola* within the same host species was found to be greater than that observed between strains from different host species. This pattern contrasts with the general trend observed in many parasites, where divergence due to isolation by host species typically exceeds that caused by isolation between the same host species (Wang et al. 2019). Phylogenetic analyses based on both SSU rDNA and ITS rDNA indicated that the strains of *C. siganicola* did not cluster according to host species nor form monophyletic clades specific to particular geographic populations (Fig. 5A, B). This phenomenon may be attributed to several factors, including the limited number of strains derived from *O. mossambicus* and the geographical distributions involved, which may not fully represent the overall divergence among strains from different host species or geographic regions. As a result, the observed greater genetic divergence among strains from the same host species and the lack of clustering based on host species or geographic origin could be influenced by sampling limitations rather than reflecting true population divergence. Therefore, future studies expanding sample sizes and host species coverage will be essential for validating the observed divergence patterns and for gaining a deeper understanding of the factors driving ceratomyxid diversification.

From the perspective of strain divergence, the divergence trends observed with different molecular markers are consistent; however, divergence based on ITS rDNA is higher than that observed with SSU rDNA (Fig. 4). This suggests that ITS rDNA is more sensitive in detecting population-level differences. The greater discriminatory power of ITS rDNA for detecting finer-scale variations among populations underscores its utility as a marker for population-level genetic studies.

## Supplementary Material

XML Treatment for Ceratomyxa dactyloptena

XML Treatment for Ceratomyxa siganicola

## ﻿Additional information

### Use of AI

No use of AI was reported.

### Author ORCIDs

Chengzhong Yang https://orcid.org/0000-0002-3850-4197

Yang Zhou https://orcid.org/0000-0002-4103-0076

Yuanjun Zhao https://orcid.org/0000-0002-0438-0747

### Data availability

All of the data that support the findings of this study are available in the main text.
